# Out-of-pocket costs for people with tuberculosis disease in Toronto, Canada: A web-based survey at two tuberculosis treatment centres, April 2023–April 2025

**DOI:** 10.14745/ccdr.v52i04a04

**Published:** 2026-04-30

**Authors:** Lauren Ramsay, Ezinne Ndukwe, Elizabeth Rea, Andrea Ackery, Jane McNamee, Thanh Nguyen, Marian Hassan, Nawang Yanga, Amrita Daftary, Kelly O’Brien, Christopher Pease, Yeva Sahakyan, Sarah Brode, Beate Sander

**Affiliations:** 1 Institute of Health Policy, Management and Evaluation, Dalla Lana School of Public Health, University of Toronto, Toronto, ON; 2 University Health Network, Toronto, ON; 3 Dalla Lana School of Public Health, University of Toronto, Toronto, ON; 4 Toronto Public Health, Toronto, ON; 5 School of Health Policy and Management, Faculty of Health, York University, North York, ON; 6 Department of Physical Therapy, Temerty Faculty of Medicine, University of Toronto, Toronto, ON; 7 Rehabilitation Sciences Institute, University of Toronto, Toronto, ON; 8 The Ottawa Hospital, Ottawa, ON; 9 Department of Medicine, University of Ottawa, Ottawa, ON; 10 Department of Medicine, University of Toronto, Toronto, ON; 11 Public Health Ontario, Toronto, ON

**Keywords:** tuberculosis, out-of-pocket costs, cost analysis, public health

## Abstract

**Background:**

In Ontario, over 600 cases of tuberculosis (TB) have been reported annually since 2015. Despite publicly funded health care, the direct and indirect costs to individuals have not been well studied and may be substantial.

**Objective:**

To assess the economic and social impacts of TB among individuals undergoing treatment at specialized TB care centers in Toronto, Canada.

**Methods:**

Patient-reported costs, work disruptions, social consequences and changes in household financial status were evaluated. A cross-sectional web-based survey was conducted among adults (18 years and older) receiving TB treatment at West Park Healthcare Centre and the Toronto Western Hospital TB clinic between April 2023 and April 2025. Respondents completed a modified version of the World Health Organization’s Tuberculosis patient cost survey, which collected data on out-of-pocket costs, work loss and social outcomes. Descriptive statistics were used to summarize key findings and reported costs in 2025 Canadian dollars.

**Results:**

A total of 42 people with TB disease completed the survey (mean age=36.7 years; 38% female). The mean cost of a TB-related appointment was $42.82 (standard deviation [SD]=$47.89), with variation by treatment center, hospitalization status and transportation method to appointments. More than half (61.9%) of respondents reported missing work due to treatment, with a mean loss of 130 hours (SD=141). Following diagnosis with TB, 28.6% of respondents experienced job loss, and 45.3% of respondents experienced a worsening of their household financial situation, with 16.7% reporting that it had become much worse.

**Conclusion:**

Despite publicly funded health care, people with TB face economic and social challenges. These findings will contribute to a deeper understanding of the financial challenges faced by people with TB and may be useful in informing public health policy.

## Introduction

Despite advances in prevention and treatment, tuberculosis (TB) continues to pose a significant public health burden globally. In Ontario, over 600 cases have been reported annually since 2015 ((1)). In Toronto, 375 people were diagnosed with TB in 2024; the highest number since 2002 ((2)). Toronto regularly records a rate of TB nearly double the provincial average. This is likely because of a higher proportion of people born outside of Canada than other parts of the province ((2)). In addition to the health consequences, people with TB often experience financial costs and adverse social outcomes, including job loss, food insecurity and housing instability, especially during the isolation period required during treatment for some people ((3)).

Canada has a publicly-funded health insurance system. In Ontario, TB-related healthcare costs are covered by provincial programs, including those not otherwise eligible for government-funded care, such as international students or visitors, via the Tuberculosis Diagnostic and Treatment Services for Uninsured Persons (TB-UP) Program ((4)); however, the financial burden experienced by individuals has not been well studied and may still be substantial.

Canada is a signatory to the United Nations Political Declaration on the Fight Against Tuberculosis, committing to eliminate catastrophic costs for all people with TB ((5)). There is a notable gap in Canadian data on individual-level TB costs; to the authors’ knowledge, no other patient cost survey has been conducted for people with TB in Canada. A survey in the Netherlands was conducted with immigrants with TB and found that patients incurred out-of-pocket costs, as well as lost days of work, despite public health insurance ((6)). Understanding patient costs related to TB is important because these costs can impede treatment adherence and are often incurred to protect public health (e.g., isolation), underscoring the societal imperative to alleviate them.

The objective of this research was to characterize out-of-pocket costs incurred by people with TB in Toronto, Ontario, Canada. This study aimed to capture patient-reported costs, including direct healthcare expenses and out-of-pocket costs as well as indirect costs such as productivity loss. The study’s findings can contribute to a deeper understanding of the financial challenges faced by people with TB and may be useful for public health policy.

## Methods

This study used a cross-sectional web-based survey between April 2023 and April 2025 to collect information from adults (18 years of age and older) undergoing treatment for TB disease. Respondents were recruited from two TB treatment centres in Toronto: West Park Healthcare Centre (WPHC) and the Toronto Western Hospital (TWH). The WPHC is a rehabilitation and complex care facility with a dedicated inpatient TB unit and outpatient clinic and is recognized as a referral centre for patients with complex TB ((7)). The WPHC became a part of the University Health Network (UHN) in April 2024, but TB program operations did not change. The TWH TB clinic is an outpatient clinic operating at the TWH, also part of UHN ((8)). The annual number of eligible patients was estimated to be approximately 85 from WPHC and 50 from TWH. This research was approved by the Research Ethics Boards at UHN (REB #22–5400) and WPHC (REB #23–001–WP).

### Survey instrument development

This study used a modified version of the World Health Organization (WHO)’s Tuberculosis patient cost survey ((9)). This standardized tool was designed to capture the economic burden experienced by people with TB and their households. Modifications were made to tailor the survey to the Ontario health care context, focusing on relevant costs and health service utilization. Key domains included in the survey instrument were direct medical costs (e.g., expenses related to hospitalization, outpatient visits, and medications), direct non-medical costs (e.g., transportation, food and accommodation related to TB care), amount of missed work, and socioeconomic and demographic characteristics.

Study data were collected and managed using REDCap electronic data capture tools hosted at UHN. The survey was offered with interpretation services in any language required by interested respondents and offered in multiple formats (in-person, online and phone) to improve survey accessibility.

### Sampling and recruitment

Clinic staff approached adults undergoing treatment for TB at the identified clinics requesting their participation in the study. Recruitment strategies were integrated into routine clinical interactions with healthcare providers. Interested respondents received detailed information about the study’s objectives, procedures and potential risks during the informed consent process prior to participation. Upon referral to the study team, contact via phone was attempted three times before assuming that the individual was no longer interested in participating in the study. The questionnaire was designed to take approximately 45 minutes to complete, and respondents were provided a $25 gift card for their participation.

### Measurement

The primary outcome measures were patient-reported costs and productivity loss associated with paid employment. Additionally, the survey instrument collected data on social outcomes based on peoples’ experiences during their treatment for TB. These outcomes included social exclusion, food insecurity, employment status and household financial security. Respondents were not provided with formal definitions for these outcomes; thus, responses reflect respondents’ personal experiences and interpretation.

### Analysis

Descriptive statistics were used to summarize respondent demographics, socioeconomic characteristics, TB-related costs, and TB-related social outcomes. Measures of central tendency (mean, median) and variability (standard deviation [SD], interquartile range [IQR]) were calculated for continuous variables, while categorical variables were summarized using frequencies and percentages.

## Results

Between the two treatment centres, 61 people were referred to the study team by their clinic staff. By April 30, 2025, 42 respondents completed the survey, representing a response rate of 68.9%.

### Baseline characteristics

The survey sample consisted of 42 respondents with a mean age of 36.7 years (SD=14.95), with a majority identifying as men (61.9%). All respondents reported being born outside of Canada (57.1% with citizenship or permanent resident status; 40.5% non-permanent residents). Over half of respondents were recruited at WPHC (57.1%). The majority had completed trade school, college, a bachelor’s degree or higher (76.1%), and approximately 75% were employed either full-time or part-time. Household income was most frequently reported in the two lowest quintiles in Canada, with over half (52.4%) of respondents’ households earning $57,700 CAD or less prior to their TB diagnosis. Almost all respondents reported some form of health insurance (including Ontario Health Insurance Plan [OHIP], TB-UP). Over half of respondents had pulmonary TB (57.1%). Characteristics of respondents are reported in **Table 1**.

**Table 1:** Baseline characteristics of survey respondents

Characteristic	n^a^	%
Sample size	42	100
Age, mean (SD) (years)	36.7 (14.95)	range: 20–71
**Gender identity**		
Man	26	61.9
Woman	16	38.1
Other^b^	0	0
**Immigration status**		
Citizen, immigrant or permanent resident	24	57.1
Citizen, born in Canada	0	0
Non-permanent resident	17	40.5
Information missing	1	2.4
**Treatment location**		
West Park Healthcare Centre	24	57.1
Toronto Western Hospital	18	42.9
**Education**		
Not completed high school or completed high school	10	23.8
Completed trade school or college	8	19.0
Bachelor’s degree	10	23.8
Post secondary above bachelor’s degree	14	33.3
**Employment status at baseline**		
Full-time	20	47.6
Part-time, casual or flex	11	26.2
Student or unemployed	11	26.2
**Household income prior to TB ($)**		
0–34,400	11	26.2
34,401–57,700	11	26.2
57,701 or higher	9	21.4
Don’t know or prefer not to answer	11	26.2
**Location of TB in body**		
Lungs	24	57.1
Outside lungs	13	31.0
Did not know	5	11.9
**Insurance status^c^**		
Provincial or federal health insurance	32	76.2
Private insurance	7	16.7
TB-UP	6	14.3
Uninsured	fewer than 6	fewer than 14.3
**Hospitalized during TB treatment**		
Yes (previously or currently)	20	47.6
No	22	52.4

Abbreviations: SD, standard deviation; TB, tuberculosis; TB-UP, treatment services for uninsured person program

^a^ Small cells (<5) were supressed throughout this table to minimize reidentification risk

^b^ This survey question also included responses for transgender man, transgender woman, non-binary or gender diverse and two spirit; however, no respondents identified these ways

^c^ Total selections can add up to more than 100% for insurance status if participants reported having more than one type of insurance coverage (e.g., private insurance and provincial health insurance). Provincial or federal health insurance included Ontario Health Insurance Plan, another provincial health insurance plan or the Interim Federal Health Program

### Outcomes

Each TB-related clinical appointment had a mean total cost of $42.82 (SD=$47.89) (**Table 2**). Costs included those related to transportation, parking and food. No respondents required childcare or accommodation to attend their routine appointments. Transportation costs for each appointment varied by mode of transportation, with the highest cost associated with respondents who traveled by taxi or rideshare at their own expense (n=17; mean cost=$70.24 per appointment) and the lowest for respondents who traveled by public transit (n=15; mean cost=$19.87 per appointment). No respondents reported any direct medical costs (e.g., medicines, appointment fees, imaging) associated with their TB care.

**Table 2:** Out-of-pocket costs (CAD) associated with one tuberculosis appointment

Sample	n	Mean (SD) ($)	Median (IQR) ($)
Overall	42	42.82 ($47.89)	29.50 ($11.25–$49.00)
**By immigration status**			
Born outside of Canada, citizen/permanent resident	24	43.65 ($49.46)	30.00 ($10.13–$44.00)
Born outside of Canada, non-permanent resident	17	43.48 ($47.96)	29.00 ($15.00–$55.00)
**By treatment centre**			
West Park Healthcare Centre	24	50.92 ($57.09)	37.00 ($10.75–$56.25)
Toronto Western Hospital	18	32.03 ($30.10)	21.75 ($12.25–$32.25)
**By travel method**			
Car	8	33.75 ($17.97)	36.00 ($27.50–$43.00)
Public transit	15	19.87 ($14.75)	16.00 ($9.00–$21.75)
Taxi or rideshare	17	70.24 ($64.21)	50.00 ($25.00–$110.00)
**By hospitalization status**			
Yes (previously or currently)	20	57.26 ($60.93)	35.00 ($6.90–$77.50)
No	22	29.71 ($27.21)	23.50 ($12.75–$7.00)

Abbreviations: CAD, Canadian dollars; IQR, interquartile range; SD, standard deviation

When stratified by immigration status, permanent residents, citizens born outside of Canada and non-permanent residents had similar costs. Costs varied by treatment centre; respondents receiving care at WPHC had a mean cost of $50.92 (SD=$57.09). Respondents receiving care at TWH had a mean cost of $32.03 (SD=$30.10). More people being treated at WPHC drove or took a taxi or rideshare to their appointments (n=18 of 24; 75.0%) compared to UHN (n=7 of 18; 38.9%). Twenty-one respondents (50.0%) indicated that they completed directly observed therapy; none reported any costs associated with this.

As a result of their TB diagnosis, 30 people (71.4%) indicated that they isolated for at least a day. Of those individuals, the IQR of number of days spent isolating was 7.8 to 30.0 days (mean=35 days; median=16.5 days).

Changes in work and household financial status following TB diagnosis varied among respondents (**Table 3**). Almost two-thirds (61.9%) missed work due to TB treatment and 28.6% experienced job loss (Table 3). Among those who lost their jobs, half were employed full-time at baseline and half were in part-time, casual or flexible roles; 66.7% were in the lowest two income quintiles. Of the 26 individuals who missed work related to their diagnosis, hours missed ranged from 6 to 480 hours, with a mean of 130 hours (SD=140.7 hours; median=80.0 hours; IQR=30.0–192.0 hours). Nearly half (45.3%) of respondent’s financial situation became somewhat or much worse, and 19.0% experienced food insecurity.

**Table 3:** Social and financial outcomes reported by people with tuberculosis

Social outcome	n^a^	%
Number of participants	42	100
Experienced job loss	12	28.6
Missed work for treatment	26	61.9
Experienced social exclusion	23	54.8
Experienced food insecurity	8	19.0
**Change in household financial status**		
Much worse	7	16.7
Somewhat worse	12	28.6
No change	more than 10	more than 23.8
Somewhat better	fewer than 6	fewer than 14.3
Much better	fewer than 6	fewer than 14.3
Missing	fewer than 6	fewer than 14.3

^a^ Small cells (<5) were supressed throughout this table to minimize reidentification risk

## Discussion

This study examined the out-of-pocket costs and social impacts of TB among people receiving treatment at two specialized TB care centres in Toronto, Canada. Key findings indicate that TB-related appointments were associated with a mean cost of $42.82 per visit. If people with TB attend 8–12 routine appointments on average, this amounts to a mean cost of $343 to $514. This study identified a difference in costs by TB treatment centre, with higher costs for WPHC compared to TWH. As a specialist referral centre, WPHC serves the Greater Toronto Area, a wide geography, and is not well served by public transit; in contrast, the TWH clinic is in downtown Toronto and has multiple transit connections.

This study also identified that 45.3% of respondents reported a decline in their household financial status following their TB diagnosis, with 16.7% indicating a substantial worsening. Among those who reported missing work, the mean hours missed was 130, which, assuming no paid leave and using the 2023 Ontario average hourly wage ($34.63), corresponds to approximately $4,502 in lost income per person. This estimate likely underrepresents the impact for individuals who lost employment entirely. Notably, nearly one-third of participants in this study reported job loss, a disproportionate share of whom (66.7%) were in the lowest two income quintiles prior to diagnosis. These findings suggest that the financial impact of job loss may be more substantial for individuals in lower-wage or precarious employment, where even short absences or disclosure of a TB diagnosis may lead to job loss.

The Canadian TB Standards provide up-to-date practical management information for the care of patients with TB including information and guidance on aspects of pathogenesis, epidemiology and TB management in Canada. It includes a practice statement that reads: “People with TB disease should be provided all medications and services required to successfully complete TB therapy free of charge, regardless of their insurance coverage or residency status in Canada,” ((10)). In this study, respondents did not report direct medical costs associated with their TB disease which is consistent with the public funding structure for TB care in Ontario ((4)). In addition to treatment and TB-related clinical care being free to people with TB in Toronto, Toronto Public Health also provides services and limited financial support to alleviate the economic burden of TB ((11)). These additional services may include social work assistance, food vouchers, paying for taxis to attend appointments and providing emergency accommodation during the mandatory isolation period. The findings of this study highlight the importance of such public health supports.

These findings align with prior research demonstrating that TB is associated with direct and indirect (productivity) costs, even in settings where most direct medical expenses are covered by publicly funded healthcare systems ((6)). While no respondents in the current survey reported direct medical costs associated with their TB care, non-medical expenses, such as transportation and productivity losses contributed to their financial burden.

### Strengths and limitations

Several limitations should be considered when interpreting these findings. The small sample size (n=42) limits the generalizability and transferability of results to other populations and settings, and the recruitment of respondents from only Toronto, an urban centre, may not be representative of the broader population of individuals with TB in Ontario or representative of the specific experiences of all migrant populations or key vulnerable populations (in 2023, 37.7% of people with TB disease in Ontario were reported by Toronto Public Health, 94% of whom were foreign-born) ((12)). The small sample size also prevented us from making associations between respondent characteristics and cost outcomes. This includes the inability to compare cost and productivity related outcomes by location of TB in the body (i.e., pulmonary or extra-pulmonary), which may impact costs due to different isolation requirements. Additionally, self-reported data on costs, work disruptions and social outcomes (which were not defined) may be subject to recall bias or social desirability bias ((13)). The cross-sectional and descriptive approach of this analysis limits the ability to determine causation between TB and patient costs. Additionally, this study was unable to compare complete responses to those who did not complete the survey. Finally, the WHO TB patient cost survey has not been formally validated in this study’s setting, and definitions for social outcomes were not provided to respondents which may reduce the reliability of responses.

Despite these limitations, this study has several strengths. The use of a modified WHO TB patient cost survey allowed for a comprehensive assessment of the financial and social impacts of TB in the Ontario health care context. Additionally, recruiting respondents from both WPHC and TWH allowed the incorporation of both complex and more standard TB care settings, improving the relevance of findings across different treatment pathways. These results contribute to a growing body of evidence on the economic and social consequences of TB and highlight the need for policies that mitigate financial hardship and support individuals affected by TB.

## Conclusion

This study found that people receiving treatment for TB in Toronto faced out-of-pocket costs and social challenges, despite publicly funded health care. These findings will contribute to a deeper understanding of the financial challenges faced by people with TB and may be useful in informing public health policy.
